# Сердечно поздравляем с 80-летием! К юбилею Леонида Григорьевича Стронгина

**Published:** 2026-05-20

**Authors:** статья Редакционная

**Affiliations:** ФГБОУ ВО "ПИМУ" Минздрава РФ Russian Federation

## Abstract

6 марта 2026 г. отмечает свой юбилей доктор медицинских наук, профессор, почетный работник высшего профессионального образования Леонид Григорьевич Стронгин — директор института терапии, профессор кафедры эндокринологии и внутренних болезней ФГБОУ ВО «Приволжского исследовательского медицинского университета» Минздрава России.

6 марта 2026 г. отмечает свой юбилей доктор медицинских наук, профессор, почетный работник высшего профессионального образования Леонид Григорьевич Стронгин — директор института терапии, профессор кафедры эндокринологии и внутренних болезней ФГБОУ ВО «Приволжского исследовательского медицинского университета» Минздрава России.

Леонид Григорьевич Стронгин — продолжатель славной научной династии. Он родился 6 марта 1946 года в городе Дзержинске Горьковской области. Дед Леонида Григорьевича, Иван Романович Брайцев (1870-1947), профессор, основатель физико-математического факультета Нижегородского госуниверситета. Отец, Григорий Михайлович Стронгин (1909–1989), — выдающийся химик, заслуженный изобретатель РСФСР, создатель незамерзающей противотанковой жидкости, дважды лауреат Сталинской премии, почетный гражданин Дзержинска.

Леонид Григорьевич Стронгин избрал путь служения науке. Окончив Горьковский медицинский институт им. С.М. Кирова с отличием в 1970 году, он начал свою деятельность как практикующий врач-терапевт. С 1971 по 1973 годы Леонид Григорьевич работал ординатором и заведующим терапевтическим отделением медсанчасти химкомбината «Корунд» в Дзержинске, а с 1976 по 1978 годы — врачом группы советских геологов в Народно-Демократической Республике Йемен.

С 1978 года его судьба неразрывно связана с Приволжским исследовательским медицинским университетом (ПИМУ), в то время Горьковским медицинским институтом им С.М. Кирова, где он прошел путь от научного сотрудника до заведующего кафедрой, проректора и директора института терапии.

Область научных интересов Леонида Григорьевича Стронгина обширна и охватывает проблемы эндокринологии, профилактики и ранней диагностики заболеваний внутренних органов, применения информационных технологий в задачах медицинской диагностики и выбора лечения. В 1981 г. Леонид Григорьевич принимал непосредственное участие в организации первого в регионе консультативного автоматизированного диагностического центра Дорожной больницы на станции Горький, в 1985 г. был ответственным исполнителем проекта автоматизированной системы «Чайка», внедренной в работу медицинской службы Байкало-Амурской железной дороги. За разработку и внедрение медицинских информационных систем в 1983 и 1984 годах награжден медалями ВДНХ. С 1991 по 1995 годы был разработчиком автоматизированной системы доврачебной диагностики МАРС, внедренной в 7 регионах России. В 1982 г. Леонид Григорьевич защитил диссертацию по лечению сахарного диабета под руководством профессора Е.П. Камышевой, в 1999 г. — докторскую диссертацию на тему: «Оптимизация диагностики и врачебной тактики в практике терапевта с использованием компьютерного моделирования».

Леонид Григорьевич Стронгин сыграл ключевую роль в развитии последипломного образования по эндокринологии в Нижегородской области. Его усилиями было организовано преподавание эндокринологии и диабетологии на кафедре терапии последипломного образования, а также создана кафедра эндокринологии и внутренних болезней с лицензированием интернатуры, ординатуры и аспирантуры по специальности «Эндокринология». Одновременно с этим, в течение двух десятилетий (1992–2012), Леонид Григорьевич возглавлял международное направление Нижегородской государственной медицинской академии, последовательно занимая должности декана факультета обучения иностранных студентов и проректора по международной деятельности. Под его началом Нижегородская государственная медицинская академия впервые открыла обучение для иностранных студентов и стала одним из пионеров в России по внедрению англоязычного медицинского образования. В 2008 г. вклад Леонида Григорьевича был отмечен премией Нижнего Новгорода «За разработку концепции преподавания на английском языке в российских вузах».

Являясь признанным клиницистом и ведущим специалистом-эндокринологом Нижнего Новгорода, Леонид Григорьевич Стронгин внес значительный вклад в организацию и развитие специализированной помощи в регионе. При его непосредственном участии на базе городской клинической больницы №13 было создано эндокринологическое отделение и открыт центр лечения тиреотоксикоза радиойодом. Также Леонид Григорьевич участвовал в создании областного тиреоидологического и городского эндокринологического центров. Леонид Григорьевич разрабатывал методическое обеспечение мобильного диабет-центра, обеспечивающего доступной медицинской помощью больных сахарным диабетом в отдаленных районах.

Результатом деятельности Леонида Григорьевича Стронгина стало формирование научной школы, изучающей патофизиологические и клинические особенности сердечно-сосудистой патологии при эндокринных заболеваниях. Публикации последних 25 лет посвящены управлению сахарным диабетом при сердечно-сосудистой патологии, коррекции гликемии в критических состояниях, поражениям нижних конечностей при сахарном диабете, терапевтическому обучению больных, патогенетическим механизмам кардиоваскулярных заболеваний при патологии щитовидной железы. Л.Г. Стронгин является автором более 400 научных работ. Под его руководством успешно защищены 25 кандидатских и 5 докторских диссертаций. Результаты научных исследований регулярно докладываются на крупнейших международных, всероссийских, межрегиональных и региональных форумах и конференциях.

Леонид Григорьевич ведет большую общественную работу, является членом редколлегий журналов «Проблемы эндокринологии», «Медицинский альманах», «Эндокринология. Новости. Мнения. Обучение», «FOCUS Эндокринология» и «Лечение и профилактика». Стронгин — почетный член организации пациентов «Нижегородская диабетическая лига». Возглавляет и входит в состав оргкомитетов многих всероссийских и межрегиональных научных форумов и образовательных мероприятий, избирался членом правления Российской ассоциации эндокринологов.

Ученики и коллеги, преподаватели кафедры эндокринологии и внутренних болезней ПИМУ желают Леониду Григорьевичу крепкого здоровья и творческого долголетия.

